# From Classroom to Clinic: A Scoping Review of Critical Thinking and Decision-Making in Orthopaedic Education for Medical Students and Residents

**DOI:** 10.1007/s12178-025-10002-x

**Published:** 2025-12-23

**Authors:** Jamie Rosen, Prerna Kartik, Martinique Vella-Baldacchino

**Affiliations:** 1 Warwick Medical School, Medical School Building, University of Warwick, Coventry, CV4 UK; 2 Kettering General Hospital, Rothwell Rd, Kettering, NN16 8UZ UK; 3 MSk Lab, Department of Surgery and Cancer, Imperial College London, 86 Wood Ln, London, W12 0BZ UK

**Keywords:** Orthopaedic education, Critical thinking, Clinical decision-making, Medical students, Orthopaedic residents, Simulation-based training

## Abstract

**Purpose of Review:**

This review examines existing literature on how orthopaedic education develops critical thinking and decision-making in medical students and residents.

**Recent Findings:**

Scopus, Web of Science, MEDLINE, and PubMed were searched for English-language studies published between 2015 and 2025. Twenty-eight studies met the inclusion criteria. Most involved residents (*n* = 19), fewer focused on medical students (*n* = 8), and one included both groups (*n* = 1). Five themes were identified: technology-enhanced learning, reflective and analytical practice, mentorship and professional development, curriculum design and integration, and assessment and feedback. Simulation and digital tools improved procedural reasoning and engagement. Reflection and mentorship supported analytical and diagnostic skills. Non-operative and outpatient decision-making were rarely explored.

**Summary:**

Orthopaedic education increasingly uses technology and active learning. However, structured development of critical thinking and decision-making remains limited, especially outside surgical settings and early training. Embedding reasoning, reflection, and mentorship in curricula may better connect classroom learning with clinical decision-making.

**Supplementary Information:**

The online version contains supplementary material available at 10.1007/s12178-025-10002-x.

## Introduction

Making the right decision, particularly in time-sensitive or uncertain scenarios, demands far more than technical skill alone [1]. It calls for the capacity to reason clearly, reflect critically, and adapt thoughtfully to the complexities of each case. Critical thinking (CT) refers to the disciplined approach to evaluating information, questioning assumptions, and drawing reasoned conclusions [2]. In clinical practice, it enables the orthopaedic learner, whether a medical student, resident, or early-career surgeon, to solve complex problems systematically, consider alternative explanations, and weigh competing options in a rational and reflective manner [3]. This process is essential not only for diagnostic reasoning but also for effective problem solving in therapeutic decision-making, particularly where uncertainty or competing priorities exist [2].

Clinical decision-making builds on these foundations. It is the process through which healthcare professionals translate clinical information into diagnostic and therapeutic choices. Effective decision-making requires the integration of knowledge, judgment, and experience, applied flexibly across a range of clinical contexts [4]. Although evidence-based guidelines and decision algorithms serve as valuable tools, they rarely account for the full complexity of individual patient presentations [5]. In orthopaedics, these challenges are particularly pronounced. Surgeons must frequently navigate choices between operative and non-operative strategies or evaluate different surgical approaches, each carrying distinct risks and benefits. Moreover, many of these decisions are made in high-pressure environments, such as the operating theatre, the emergency department, or trauma resuscitation settings, where judgment must be applied quickly [6].

Although it is well-established that CT and decision-making are crucial in surgical practice, there is still inconsistency in the structured development of these cognitive abilities in orthopaedic education. Technical proficiency has always been the prioritised in surgical training, whilst clinical exposure and experience was thought to implicitly develop thinking and judgment [7]. However, studies in medical education increasingly challenge this assumption. There is growing evidence that reasoning and decision-making skills can be taught explicitly and that deliberate educational strategies may improve these skills [5].

Despite this, it remains unclear how these approaches have been applied to support cognitive development within orthopaedic education specifically. This scoping review evaluates educational interventions reported over the past 11 years that aim to develop CT and DM among medical learners, spanning pre-clinical students to the most advanced levels of orthopaedic training, within contemporary academic and clinical learning environments.

## Methods

The protocol was drafted using Preferred Reporting Items for Systematic Reviews and Meta Analysis extension for Scoping Reviews (PRISMA-ScR) [8]. A literature search was performed using the following databased: Scopus, Web of Science, MEDLINE and PUBMED for published articles. The inclusion criteria consisted of peer-reviewed studies published 2015 to 2025, in English, that examined the effects of educational interventions or thought processes on CT and decision-making amongst medical students and orthopaedic registrars. The search spanned the past eleven years to capture current educational practices and recent technological developments in medical training. This timeframe was considered suitable given the limited evidence on CT and DM in orthopaedic education, and older studies were included only where they offered relevant insight. The exclusion criteria consist of studies not in English, published over eleven years, letters to the editor. The full eligibility criteria are expressed in Table 1.

**Table 1:** PICO (Inclusion and Exclusion Criteria)

Population	Medical Students and Orthopaedic Resident Doctors
Intervention	Educational activities, Clinical placement, Decision-making tasks that require students to apply reasoning, solve diagnostic or management problems, or reflect on patient cases.
Comparison	Can include comparison between forms of teaching methods.
Outcome	Demonstrated or self-reported development of clinical reasoning, diagnostic accuracy, decision-making confidence, use of structured thinking frameworks.
Study Design	Original research and review articles published in English between 2015 and 2025. Includes studies in clinical or educational settings. Excludes opinion pieces, editorials, non-peer-reviewed sources, case reports, and grey literature.

The search strategy included both text words and Medical Subject Headings (MeSH)/thesaurus headings terms and a range of keywords such as “Medical Student”, “Decision-Making”, “Resident”, “Orthopaedics” and “Critical thinking” were used. Boolean operator (AND/OR) were applied to ensure optimal retrieval of relevant literature. Appendix 1 demonstrates the search string used. Two authors independently reviewed all studies identified in the initial search based on their titles and abstracts. The studies were classified as “Include” and “Exclude”. In cases where further classification was required, an independent third author resolved any studies classified as “Maybe”. After analysis of full text articles, the selected studies were extracted into a Microsoft Excel Spreadsheet. The authors name, year that it was published, the type of intervention used and the verdict on its effectiveness were extracted.

The extracted data were subjected to thematic analysis following the six-step approach described by Braun and Clarke [9]. Two reviewers independently coded the extracted text, focusing on descriptions of interventions, study aims, and reported outcomes related to CT and clinical decision-making, with discrepancies resolved by consensus or through a third reviewer when required. Codes were collated into broader categories and iteratively refined into overarching themes, using both inductive generation from the data and deductive guidance from established concepts in medical education. This process resulted in five themes: Technology-Enhanced Learning, reflecting the use of simulation, digital platforms, and AI-based tools; Reflective and Analytical Practice, capturing interventions that promoted critical self-appraisal and problem-solving; Mentorship and Professional Development, encompassing supervision, feedback, and role-modelling; Curriculum Design and Integration, referring to structured incorporation of decision-making training within programmes; and Assessment and Feedback, addressing evaluation methods used to measure or reinforce CT. Each theme was explicitly linked back to the reviewed studies by charting interventions and outcomes within the thematic framework.

In line with recommendations for evaluating educational interventions, we undertook a basic appraisal of methodological quality using the Medical Education Research Study Quality Instrument (MERSQI) [10]. The MERSQI was applied to eligible intervention studies to assess the rigour of study design, data collection methods, and outcome measurement.

## Results

**Fig. 1:**
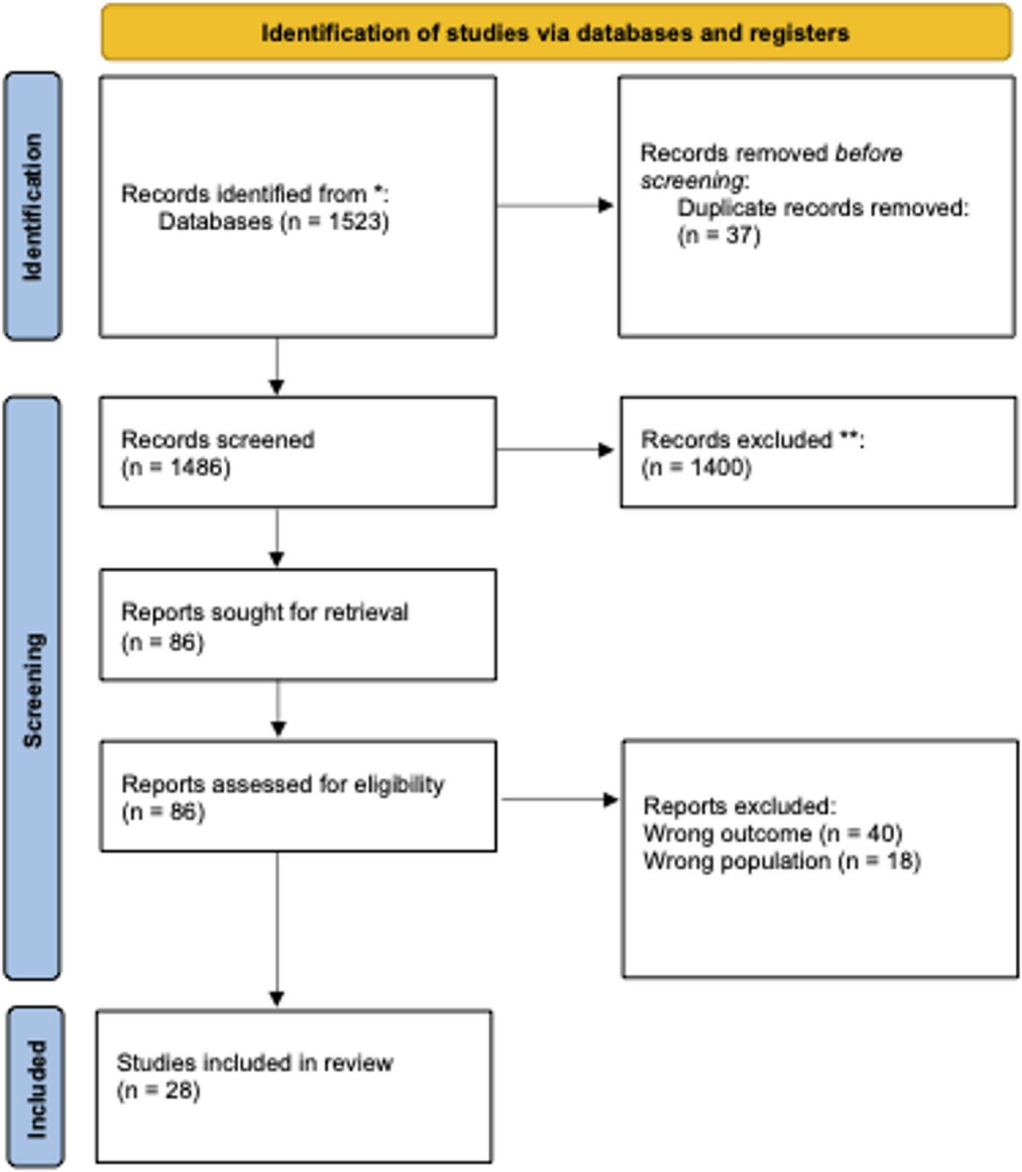
PRISMA flow diagram illustrating study selection process, including the number of records identified, screened, excluded, and included in the final review

**Fig. 2:**
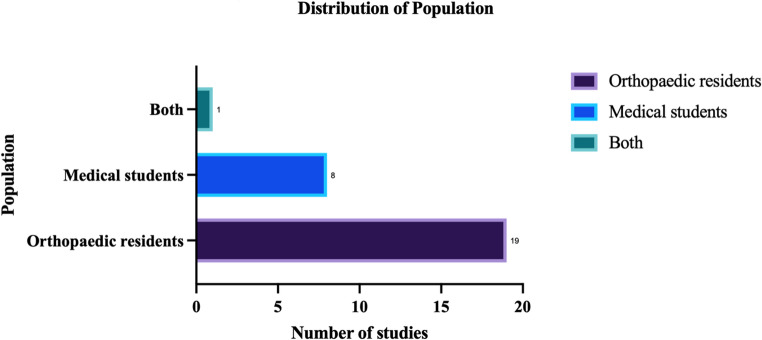
Distribution of population. The majority of studies looked at orthopaedic residents (n = 19), followed by medical students (n = 8). One study involving both orthopaedic residents and medical students (n=1)

A total of 1523 studies fit were extracted from 4 databases, of which 37 duplicates were removed. An additional 1400 studies failed to meet the inclusion criteria were removed through headings and abstract analysis. A full-text analysis was conducted on 86 remaining studies, of which only 28 met the inclusion criteria. Results of the search are represented in the PRISMA Flow Diagram (Fig. 1):

The included studies are summarised (Table 2), outlining author, year, study design, population, intervention, and key findings. For clarity, the studies are grouped into five thematic domains: assessment and feedback, curriculum design and integration, mentorship and professional development, reflective and analytical practice, and technology-enhanced learning. The distribution of studies by learner group is shown (Fig. 2).

**Table 2:** Study demographics and key findings

Author	Year	Study Design	Population	Population Size	Country	Theme	Intervention	Key Findings
Agarwal et al.[11]	2025	RCT^1^	Medical students	105	India	Technology-Enhanced Learning	3D Models	3D-model group scored higher in clinical reasoning (*p* = 0.015); 92% reported better fracture anatomy understanding and requested more sessions.
Aiyer et al.[12]	2021	Review	Medical students	-	USA	Mentorship and Professional Development	Mentorship framework	Mentorship is vital in orthopaedics; structured guidance can address inequities and support student
Al-Hubaishi et al.[13]	2023	Cohort	Orthopaedic residents	5	Canada	Assessment and Feedback	Video-based skill assessment	Video review enabled reliable performance ratings, promoted detailed feedback, and supported critical thinking through reflective skill analysis.
Alomar et al.[14]	2022	Cross-sectional	Medical students	350	Saudi Arabia	Assessment and Feedback	Mini-CEX	Mini-CEX was perceived as valuable for clinical exam prep and judgment; limited by poor instructor engagement and feedback.
Ariyana et al.[15]	2019	Observational	Medical students	24	Australia	Technology Enhanced Learning	Virtual reality simulator	VR arthroscopy training improves technical skills with practice; the steep learning curve fosters critical thinking and procedural decision making.
Atesok et al.[16]	2019	Review	Both	-	USA	Curriculum Design and Integration	Simulation curriculum review	Effective simulation integration requires validated tools, clear objectives, and inclusion of decision making to improve training quality.
Bae. [17]	2015	Review	Orthopaedic residents	-	USA	Technology Enhanced Learning	Surgical simulation	Simulation improves skills, decision making, and preparedness in paediatric orthopaedics.
Bhattacharyya et al.[18]	2017	RCT	Orthopaedic resident	3	UK	Reflective and Analytical Practice	Cognitive task analysis tool (CTA)	CTA training significantly improved procedural understanding and critical thinking in surgical steps compared to standard guidance.
Casey et al.[19]	2023	Review	Orthopaedic residents	-	USA	Assessment and Feedback	Video and motion analysis	Supports surgical training by enhancing critical thinking and decision making through objective feedback.
Chaves et al.[20]	2022	RCT	Orthopaedic residents	79	Brazil	Reflective and Analytical Practice	Debiasing through reflection	Deliberate reflection improved diagnostic accuracy by reducing confirmation bias, enhancing decision making and critical thinking.
Daurka et al.[21]	2015	RCT	Orthopaedic residents	20	UK	Reflective and Analytical Practice	Algorithm training (pelvic trauma)	Structured ABC algorithm improved emergency decision making, timing, and critical thinking in trauma simulations.
Drouaud et al.[22]	2024	Cross-sectional	Orthopaedic residents	4	USA	Technology Enhanced Learning	AI tool assessment	ChatGPT-4 V showed moderate performance; useful for management reasoning but not suitable as a standalone educational tool.
Enson et al.[23]	2022	Review	Orthopaedic residents	-	UK	Mentorship and Professional Development	Mentorship in training	Mentorship improves trainee development, supporting critical thinking, decision making, and academic performance.
Gross et al. [24]	2024	Review	Medical students	-	USA	Reflective and Analytical Practice	Art-based anatomy learning	Art analysis improved anatomical understanding and critical thinking; students valued it as an engaging learning tool.
Hiemstra et al.[25]	2024	Cross-sectional	Orthopaedic residents	43	Canada	Technology Enhanced Learning	Immersive VR demonstration	Surgeons and fellows strongly endorsed immersive VR for education, planning, and mentoring; perceived as valuable for decision making and critical thinking.
Hooda et al.[26]	2020	Cross-sectional	Orthopaedic residents	132	India	Curriculum Design and Integration	Residency Review	Strong operative exposure: gaps in structured teaching and feedback hindered development of decision-making skills.
Iramaneerat et al. [39]	2023	Cross-sectional	Orthopaedic residents	48	Thailand	Reflective and Analytical Practice	Learning style analysis	Assimilating/Converging learners trended toward higher scores; reflective strategies may aid learning.
Klima et al.[27]	2017	Cross-sectional	Medical students	237	Germany, New Zealand	Curriculum Design and Integration	Integrated anatomy–orthopaedics course	Integrated teaching improved clinical understanding, critical thinking, and student interest in orthopaedics as a career.
Kuhn et al.[28]	2024	Cross-sectional	Orthopaedic residents	6	USA	Technology Enhanced Learning	Virtual reality training	Residents found VR useful for early training; barriers include access, time, and faculty support for integration.
Lensing G et al.[29]	2022	Cross-sectional	Orthopaedic residents	103	USA	Curriculum Design and Integration	Learning style comparison	Aligning teaching with learner preferences can enhance engagement, critical thinking, and decision making in orthopaedic training.
Logishetty et al.[30]	2018	RCT	Medical students	24	UK	Technology Enhanced Learning	Augmented reality training	AR training reduced placement error vs. expert coaching; trainees valued AR as an effective and preferred adjunct to learning.
Mendes Júnior et al.[31]	2021	Cohort	Orthopaedic residents	56	Brazil	Mentorship and Professional Development	Formal mentorship program	Structured mentorship improved resident preparation, confidence, and supported critical thinking and decision making.
Phillips et al.[32]	2016	Observational	Orthopaedic residents	63	USA	Assessment and Feedback	Direct observation assessment	Structured observation enabled real-time feedback on communication, decision making, and critical thinking; supported individualized progression tracking.
Rouleau et al.[33]	2016	Observational	Orthopaedic residents	60	Canada	Curriculum Design and Integration	Intensive surgical course	National shoulder course improved knowledge and clinical decision making; effective multi-centre subspecialty training.
St Mart et al.[34]	2023	Review	Orthopaedic residents	-	UK	Technology Enhanced Learning	Artificial intelligence in orthopaedics	Trainees must develop familiarity with AI to support decision making and critical thinking.
Teng et al.[35]	2025	Cross-sectional	Medical students	40	China	Technology Enhanced Learning	Virtual reality trauma training	VR training improved clinical reasoning, technical skills, and decision making in pelvic trauma; well-received by students and faculty.
Watrinet et al.[36]	2024	Cross-sectional	Orthopaedic residents	115	Austria, Germany, Switzerland	Mentorship and Professional Development	Mentorship presence vs. absence	Residents with mentors reported stronger skills, more confidence in independent surgery, and better decision making; mentorship is widely desired.
Yang et al.[37]	2019	Cohort	Orthopaedic residents	12	USA	Assessment and Feedback	Post-call case review program	Structured autonomy with feedback reduced decision and technical errors; supported clinical decision making while maintaining patient safety.

^1^Randomised control trial

## Technology-Enhanced Learning

Nine studies evaluated virtual reality (VR), augmented reality (AR), artificial intelligence (AI), 3D printing and video-based. VR trauma simulations improved procedural reasoning. In a pelvic injury module, students demonstrated significant gains in managing haemorrhagic shock and applying external fixation (*p* < 0.001) [35]. VR arthroscopy simulators enhanced psychomotor skills, with mean task time reducing from 423 s to 242 s (− 42.7%) [15]. AR guidance in total hip arthroplasty reduced implant orientation error (6° vs. 1° with feedback), achieving accuracy comparable to expert-taught learners [30]. Immersive VR surveys showed strong endorsement, with 78–98% agreement for teaching and 88–100% for remote mentoring [25]. Resident interviews confirmed VR’s utility for anatomy review and rehearsal but noted barriers such as access, cost and technical support, with some preferring videos [28]. AI tools such as GPT-4 V demonstrated promise for reasoning (management 3.76/5; treatment 4.04/5) but lower performance in image interpretation (3.46/5) [22, 34]. Three-dimensional printing also enhanced fracture model–based learning in undergraduates [11]. A review in paediatric orthopaedics identified simulation as effective for improving technical skills and reducing errors [17]. Overall, technology-enhanced learning consistently supported improvements in accuracy, clinical reasoning, and learner engagement, although the extent of benefit differed between modalities.

## Reflective and Analytical Practice

Reflective and analytical strategies were widely used to improve decision-making in orthopaedic education. Dual-process reasoning underpins many of these approaches. It distinguishes between fast, intuitive “Type 1” and slower, analytical “Type 2” processes. Reflection-based learning aims to activate Type 2 reasoning by prompting learners to compare alternative diagnoses and seek disconfirming evidence. In a study of 79 third-year residents, reflection reduced confirmation of misleading referral diagnoses (25.9% vs. 17.6%, *p* = 0.003) and improved accuracy when cues were correct (62.0% vs. 49.1%, *p* = 0.021) [20]. Outcomes were measured using written diagnostic case vignettes.

Cognitive task analysis (CTA) promotes analytical practice by breaking procedures into defined steps, decisions, and common pitfalls. In a randomised trial of 16 residents, CTA based training improved knee arthroscopy performance, with higher Arthroscopic Surgical.

Skill Evaluation Tool (ASSET) ratings (19.5 vs. 10.6, *p* = 0.002) and task-completion scores (10.4 vs. 3.3, *p* < 0.001) [18]. Structured frameworks had similar effects. In 20 orthopaedic trainees, an ABC structured approach improved pelvic trauma management, with higher scores in observed in coagulopathy (*p* < 0.001) and prioritisation (*p* = 0.006) [21].

Reflective practice was also analysed through other educational approaches. Kolb learning-style analysis, which classifies learners into four experiential styles (assimilating, converging, diverging, and accommodating), was studied in 48 residents [38]. The study observed no significant performance differences, although assimilating and converging types scored above average [39]. Arts-based anatomy teaching using Renaissance artworks further enhanced observation and critical thinking in medical students [24]. These interventions support reflection and analysis as drivers of diagnostic accuracy and decision quality in orthopaedic training [18, 20, 21, 24, 39].

## Mentorship and Professional Development

Mentorship plays a central role in orthopaedic education, improving both clinical and academic development of orthopaedic residents and medical students. A study of Austrian, German and Swiss residents observed that those with mentors rated their theoretical and practical skills higher and felt less anxiety when operating unsupervised. They also reported better team integration and greater research output [36]. In a Brazilian orthopaedic residency’s formal mentorship program, 96% recommended the program, and 89% reported mentor influence on personal and professional decision-making [31]. A systematic review on the impact of mentoring in orthopaedic training for residents and medical students observed that mentorship influences career choice, research productivity, and mental health. The study concluded that mentorship programmes were associated with higher satisfaction students and residents [23]. For medical students, mentorship increased exposure to orthopaedics and supported career decision-making through structured guidance and feedback. It also promoted CT by encouraging reflection, goal setting, and engagement in research. This improved both research output and educational performance in medical students [12]. Confidence, reflection, and academic engagement gained through mentorship provide a foundation for critical thinking and decision-making in orthopaedic education.

## Curriculum Design and Integration

Five studies examined how curricula design and integration improved engagement and clinical reasoning. One study described an anatomy course that incorporated biomechanics, dissection, surgical approaches, and sports medicine. Medical students reported higher engagement and understanding, with surveys highlighting improvement in critical thinking and clinical and decision making [27]. A survey of 132 orthopaedic residents from the Post-Graduate Institute of Medical Sciences and Research (PGIMER) residency found that 97% of residents rated their training positively. Structured rotations, graded autonomy, and mentorship were identified as central to preparing residents for independent surgical planning and rapid decision-making in both trauma and elective settings [40]. A study of 103 residents assessed learning styles using the Kolb Learning Style Inventory. The study found “Deciding,” “Acting,” and “Thinking” to be the dominant learning styles, reflecting structured reasoning and goal-directed problem solving. Aligning curricula with these preferences may help support clinical decision-making [29].

Another study conducted a national shoulder course for 60 residents which combined cadaveric practice, case-based learning, and didactic teaching. Satisfaction exceeded 90%, and participants reported improved diagnostic reasoning [33]. A review of simulation-based orthopaedic surgical skills training reported that scenarios provided opportunities to practise critical thinking and decision-making. Evidence suggests that these skills transferred to real operating theatre settings [16]. These findings suggest that integrated curriculum can help build the critical thinking and decision-making skills needed throughout orthopaedic education.

## Assessment and Feedback

Five studies investigated assessment and feedback strategies in orthopaedic education. Five studies examined assessment and feedback in orthopaedic training. The mini-CEX was evaluated in 350 undergraduates during outpatient rotations. Students reported gains in clinical judgment, examination skills and Objective Structured Clinical Examination (OSCE) preparation. However, they noted that limited faculty engagement reduced the quality of the feedback they received [14]. In residency clinics, direct observation checklists were used during patient encounters to assess communication, professionalism and reasoning. Immediate feedback from structured ratings identified residents who needed extra support and enabled tracking of progress over time [32]. A paediatric trauma program introduced daily post-call case review of 1,298 fracture cases. Decision-making errors reduced from 3.1% to 2.3% and technical errors from 9.1% to 7.3%, while complication rates remained stable. This suggests that structured review improved decision-making without compromising safety [37].

Simulation-based approaches added further value. Video-based assessment of cadaveric fixation procedures showed strong inter-rater reliability (Interclass Correlation Coefficient = 0.83) and facilitated structured debriefing and repeated review [13]. A synthesis of film review and motion analysis reported mixed effects on learning outcomes but consistent utility for differentiating experience levels and tracking skill progression [19]. Collectively, these approaches demonstrate that structured assessment and feedback enhance both technical and non-technical skills in orthopaedic training.

## Discussion

This scoping review identified 28 studies evaluating educational interventions aimed at enhancing CT and decision-making amongst medical students and orthopaedic residents. The findings reveal several important trends and gaps in the current literature.

A disproportionate number of studies focused on orthopaedic residents compared to medical students. While this may reflect the clinical complexity and autonomy expected at the residency level, it leaves a notable gap in early-stage educational strategies. CT skills begin developing during undergraduate training, and limited attention to medical students may represent a missed opportunity to build up clinical reasoning earlier in the educational continuum. Future work should explore how foundational skills in critical analysis and decision-making can be nurtured in pre-clinical and early clinical education.

Most included studies emphasized interventions targeting technical or procedural skill development, often using simulation-based tools like VR, AR, or 3D printing. While these technologies undoubtedly enhance procedural accuracy and confidence, their focus often rests more on motor skills and task execution than on cognitive decision processes. The dominance of surgical simulation highlights a bias in orthopaedic education research toward observable technical performance, potentially at the expense of cultivating deeper diagnostic reasoning, prioritization, and judgment skills.

Similarly, decision-making was overwhelmingly addressed in the context of operative or perioperative planning, such as implant choice, fracture stabilization, or procedural steps. Very few studies focused on broader clinical decision-making like differential diagnosis, non-surgical management, or longitudinal care planning. This gap is significant, particularly as orthopaedic care increasingly incorporates shared decision-making, evidence-based practice, and patient-centred care models. Interventions aimed at improving non-operative decision-making, particularly in outpatient and emergency contexts, are needed.

Mentorship emerged as a consistent theme across several studies, with strong associations between formal mentoring structures and improved resident confidence, academic productivity, and readiness for independent practice. Importantly, residents with mentors reported less anxiety, greater self-efficacy, and more engagement with scholarly work. Despite variability in mentorship models, none showed harm, and nearly all highlighted the value of accessible, structured guidance. Given the growing interest in professional identity formation and clinician-scientist development, formal mentorship programs appear not only beneficial but essential for orthopaedic training.

Systematic reviews and primary studies show growing interest in active learning strategies to enhance CT and decision-making in orthopaedic training. For example, one paper reviewed 51 randomized trials of problem-based learning (PBL) in orthopaedic courses [41]. They found PBL significantly increased residents’ knowledge, procedural and clinical skill scores, and student satisfaction versus traditional lectures. Importantly, PBL also improved higher-order thinking: learners using PBL showed better CT, problem-solving and communication skills compared to conventional teaching. Similarly, simulation-based education is shown to develop decision-making competence [42]. A 2025 review reported that orthopaedic simulators reduced surgical errors and shortened learning curves, indicating better skill acquisition and decision-making under pressure [43]. They conclude simulation will modernise orthopaedic education by giving trainees a safe, repeatable practice that builds both technical and cognitive skills.

Despite ongoing advances in medical education, evidence suggests that residents’ critical thinking (CT) abilities frequently remain at a moderate level of proficiency. In one study conducted at Tehran University, the California Critical Thinking Skills Test (CCTST) revealed mean scores of 13.8 out of 34, placing most residents within the ‘Moderate’ performance band, below the threshold typically associated with strong or proficient critical thinking [44]. Although an improvement was observed in Postgraduate Year 2 (PGY2), a decline in performance during the later years of training (PGY3–4) raises concerns about the sustainability of CT skill development over time. Notably, orthopaedic residents scored significantly lower than their internal medicine counterparts (mean 13.1 vs. 14.5, *p* = 0.021), suggesting potential specialty-specific disparities in opportunities to engage in structured reasoning and reflective decision-making. These findings underscore the need for more targeted and longitudinal educational strategies to support the cultivation of higher order thinking skills throughout residency training.

To address this, educators recommend integrating explicit CT training. For instance, a study noted that engaging orthopaedic trainees in research projects enhances their CT and evidence-based reasoning [45]. Likewise, curriculum reviews urge the use of cognitive task analysis and problem-oriented approaches in orthopaedics so that trainees learn why each decision is made. Analyses of the shoulder and elbow domain of the Orthopaedic In-Training Examination (OITE) have consistently shown a substantial proportion of questions targeting clinical management and decision-making rather than simple factual recall. In one analysis, 45% of questions were related to treatment modalities, while a subsequent study found that 39% were classified as evaluation/decision-making (taxonomy T3) [46, 47]. These findings highlight a sustained emphasis within this subspecialty assessment on higher-order reasoning and clinical judgment over rote knowledge.

Broader surgical education literature echoes these findings. Simulation and team-based training are repeatedly cited as key for developing surgical judgment. Another study emphasises that advanced surgical simulators expose trainees to complex scenarios, helping them cultivate “critical thinking and decision-making abilities” needed for intricate cases [48]. Likewise, a study in 2019 reviewed methods for teaching non-technical surgical (NTS) skills. They found that high- and low-fidelity simulations are among the most evidence-based modalities for training these cognitive skills [49]. It was found that deficiencies in NTS, including communication, teamwork, situational awareness and DM, are significant contributors to surgical error. On this basis, the incorporation of structured NTS training into surgical curricula was recommended, with particular emphasis on simulation-based scenarios accompanied by debriefing [49].

The MERSQI score of each applicable study was calculated in order to evaluate the quality of studies. Full scores for papers can be seen in Table 3.

**Table 3:** MERSQI score for included studies

Author	MERSQI score (/18)
Agarwal et al.[11]	12.5
Aiyer et al.[12]	N/A
Al-Hubaishi et al.[13]	16.5
Alomar et al.[14]	9.5
Ariyana et al.[15]	14.5
Atesok et al.[16]	N/A
Bae. [17]	N/A
Bhattacharyya et al.[18]	16
Casey et al.[19]	N/A
Chaves et al.[20]	9.5
Daurka et al.[21]	16
Drouaud et al.[22]	8.5
Enson et al.[23]	N/A
Gross et al. [24]	N/A
Hiemstra et al.[25]	9.5
Hooda et al.[26]	11
Iramaneerat et al. [39]	9.5
Klima et al.[27]	9.5
Kuhn et al.[28]	9
Lensing G et al.[29]	9.5
Logishetty et al.[30]	12.5
Mendes Júnior et al.[31]	10
Phillips et al.[32]	12
Rouleau et al.[33]	10
St Mart et al.[34]	N/A
Teng et al.[35]	11
Watrinet et al.[36]	11.5
Yang et al.[37]	14.5

Across the 28 studies, 18 (64.3%) were published after 2019, suggesting that interest in CT and DM within orthopaedic education has only recently gained momentum. Much of this work focussed on technology-based interventions, with fewer studies addressing mentorship, curriculum design or assessment. The quality of the available evidence was mixed. Where MERSQI scoring was possible, values ranged from 8.5 to 16.5 out of 18. Some studies showed stronger methodological design, while others relied on small cohorts or self-reported outcomes. Existing literature has evolved but still contains clear gaps in both the amount of research available and the robustness of its methods.

This review is, to our knowledge, the first to map the literature on CT and decision-making in orthopaedic education. By synthesising findings across 28 studies, it highlights clear gaps in undergraduate training, an overemphasis on simulation-based technical skill development at the expense of cognitive reasoning, and the limited exploration of non-operative decision-making. The thematic framework generated from this review provides a structured lens through which future educational strategies can be developed, ensuring that orthopaedic training fosters not only procedural competence but also the higher-order reasoning essential for modern clinical practice.

This scoping review has several limitations that should be acknowledged. First, there was considerable heterogeneity among the included studies in terms of design, population, and outcome measures, which limited direct comparisons and precluded meta-analysis. While thematic analysis allowed for the synthesis of broad patterns, the diversity of methodologies and inconsistent definitions of “critical thinking” and “decision-making” made it challenging to assess intervention effectiveness systematically. Second, the literature disproportionately focused on surgical and simulation-based decision-making, often emphasizing procedural accuracy over cognitive or clinical reasoning. This reflects a skew in the current evidence base toward technical skill development, with relatively limited attention to non-surgical decision-making, such as diagnostic reasoning or outpatient management. The majority of studies were conducted among orthopaedic residents, with fewer studies addressing medical students, potentially overlooking earlier opportunities to develop CT skills. The lack of standardized measurement tools for assessing CT limited the consistency and generalizability of reported outcomes. Additionally, only peer-reviewed studies published in English were included, which may have introduced publication and language bias. Finally, the thematic analysis was conducted manually, based on a close reading of the study summaries. While this allowed for thoughtful interpretation, it may have introduced subjectivity and limited the precision of how frequently each theme appeared across studies.

Future directions could include the exploration of blended learning models that integrate simulation with case-based discussions, allowing trainees to apply both technical and cognitive skills in realistic scenarios. Institutions might consider piloting formal mentorship schemes with a focus on decision-making support, as well as embedding structured reflection exercises into clinical rotations. There is also potential to develop digital platforms or mobile applications that guide learners through critical reasoning pathways during clinical encounters. In undergraduate settings, early exposure to diagnostic uncertainty and decision-making dilemmas through problem-based learning modules or interactive vignettes could provide a valuable foundation. Future studies could also investigate how AI tools such as GPT-4 V might be adapted to support training in clinical judgment, even if their current diagnostic accuracy is limited.

## Conclusion

The findings of this scoping review underscore a clear shift toward incorporating active, technology-enhanced strategies in orthopaedic education, particularly for surgical skill acquisition. However, CT and decision-making, especially outside of the operative setting, remain underrepresented and under-assessed. Future research should focus on developing standardized definitions and validated tools to assess CT in both undergraduate and postgraduate contexts. Moreover, greater inclusion of medical students and attention to cognitive strategies, such as structured reflection and awareness of one’s thinking process, could help foster earlier and more robust reasoning skills. Expanding structured mentorship, promoting reflective practice, and embedding cognitive decision-making into curricula will be essential steps forward. In conclusion, although progress has been made, there is still a clear need to better connect technical skills training with the development of CT and decision-making in orthopaedic education.

## Key References

- Teng P, Xu Y, Qian K, Lu M, Hu J. Case-Based Virtual Reality Simulation for Severe Pelvic Trauma Clinical Skill Training in Medical Students: Design and Pilot Study JMIR Med Educ 2025;11:e59850.
  - This manuscript demonstrates that VR pelvic-trauma simulation significantly improved procedural reasoning and management accuracy, highlighting simulation’s role in enhancing orthopaedic decision-making.
- Bhattacharyya R, Davidson DJ, Sugand K, Bartlett MJ, Bhattacharya R, Gupte CM. Knee Arthroscopy Simulation. Journal of Bone and Joint Surgery. 2017;4;99(19):e103.
  - This manuscript highlights that cognitive-task-analysis training improves arthroscopic performance, evidencing that structured analytical methods strengthen clinical reasoning.
- Enson J, Malik-Tabassum K, Faria A, Faria G, Gill K, Rogers B. The impact of mentoring in trauma and orthopaedic training: a systematic review. The Annals of The Royal College of Surgeons of England. 2022;104(6):400–8.
  - This manuscript found that formal mentorship in orthopaedic training improves residents’ confidence, judgment, and academic output, reinforcing its value for independent decision-making.
- Hooda A, Dhillon MS, Neradi D, Kumar D, Vatsya P, Shetty A. Orthopedic Residency in a Tertiary Care Hospital of India: Positives, Negatives and Perspectives for Change. Indian Journal of Orthopaedics. 2021;55(S1):209–16.
  - This manuscript reported that structured rotations, graded autonomy, and mentorship improve decision-making readiness, underscoring the impact of curriculum design on cognitive development.
- Yang BW, Waters PM. Implementation of an Orthopedic Trauma Program to Safely Promote Resident Autonomy. Journal of Graduate Medical Education. 2019;11(2):207–13.
  - This manuscript showed that structured feedback reduced decision-making errors, confirming that systematic review processes enhance reasoning and safety in orthopaedic practice.

## Supplementary Information

Below is the link to the electronic supplementary material.

Supplementary Material 1 (DOCX 15.5 KB)

## Data Availability

No datasets were generated or analysed during the current study.
