# Supplementary material for: From Classroom to Clinic: A Scoping Review of Critical Thinking and Decision-Making in Orthopaedic Education for Medical Students and Residents
Source: Curr Rev Musculoskelet Med. 2025 Dec 23;19(1):9. doi: 10.1007/s12178-025-10002-x (PMC12722595; doi:10.1007/s12178-025-10002-x)
Supplement: Supplementary file 1 — Supplementary Material 1 (DOCX 15.5 KB) [file 12178_2025_10002_MOESM1_ESM.docx]

**Appendix**

**Search String**

TITLE ( "orthopaedic" OR "orthopedic" OR "orthopaedic surgery" OR "orthopaedic surgeon" OR "trauma surgery" ) AND ( TITLE-ABS-KEY ( "orthopaedic resident" OR "orthopedic resident" OR "medical student" OR "orthopaedic trainee" OR "surgical trainee" ) ) AND ( TITLE-ABS-KEY ( "critical thinking" OR "clinical reasoning" OR "clinical judgement" OR "decision-making" OR "problem solving" OR "diagnostic reasoning" OR "situational awareness" OR "reflective practice" OR "reflection" OR "metacognition" OR "cognitive bias" OR "heuristics" OR "risk assessment" OR "evidence-based practice" OR "shared decision making" OR "judgment" OR "non-technical skills" OR "human factors" OR "communication skills" OR "teamwork" OR "leadership" OR "mentorship" OR "coaching" ) )

AND ( TITLE-ABS-KEY ( "education" OR "training" OR "curriculum" OR "teaching" OR "simulation" OR "feedback" OR "virtual reality" OR "augmented reality" OR "assessment" OR "competency" OR "competence" ) )AND ( LIMIT-TO ( LANGUAGE , "English" ) )

AND ( LIMIT-TO ( EXACTKEYWORD , "orthopedic surgery" ) OR LIMIT-TO ( EXACTKEYWORD , "orthopedics" ) )AND ( LIMIT-TO ( PUBSTAGE , "final" ) )

AND ( LIMIT-TO ( DOCTYPE , "ar" ) OR LIMIT-TO ( DOCTYPE , "re" ) )AND ( LIMIT-TO ( SRCTYPE , "j" ) )AND ( LIMIT-TO ( PUBYEAR , 2015-3000 ) )
